# The translation and psychometrics Persian version of irrational food belief scale

**DOI:** 10.1186/s12888-023-04909-3

**Published:** 2023-06-15

**Authors:** Fatemeh Afsahi, Mansoor Alimehdi, Hamid Sharif-Nia

**Affiliations:** 1 Department of Psychology, Islamic Azad University, Tehran Medical Sciences Branch, Tehran, Iran; 2 Department of Psychology, Faculty of Medicine, Islamic Azad University, Tehran Medical Branch, Tehran, Iran; 3 Tehran Islamic Azad University of Medical Sciences, Building Intersection of Karimi St. Shahid Mosivand St. Shariati St., Tehran, 1916893813 Iran; 4 Traditional and Complementary Medicine Research Center, Addiction Institute Mazandaran University of Medical Sciences, Sari, Iran; 5 Department of Nursing, Amol Faculty of Nursing and Midwifery, Mazandaran University of Medical Sciences, Sari, Iran

**Keywords:** Translations, Psychometrics, Food beliefs, Irrational beliefs, Validation study

## Abstract

**Background:**

This study aimed to translate into the Irrational Food Belief Scale proposed by Osberg into the Persian language and determine the psychometric properties of in Iranian culture.

**Methods:**

Osberg’s 57-item scale was translated into Persian by using the forward-backward method. The scale’s validity was examined using face validity, content validity, and construct validity (exploratory and confirmatory factor analysis). Its reliability was assessed with Cronbach’s alpha and McDonald’s Omega coefficient. Exploratory factor analysis and confirmatory factor analysis was performed by SPSS 28 (500 subjects) and also by AMOS 26 (500 subjects). The participants completed the demographic questionnaire and the Irrational Food Belief Scale (IFBS) over the Internet.

**Results:**

After translation into Persian, the validity of the scale was determined by impact score, quantitative and qualitative face validity (modification of 10 items) and qualitative content validity (modification of 8 items), and quantitative content validity (CVR, CVI and Kappa coefficient), which were greater than 0.46, 0.86, and 0.85, respectively. In exploratory factor analysis, 30 items were removed and the remaining 27 items were loaded on five factors, including behavioral and psychological aspects, nutritional attitudes, healthy eating, controlled eating, and diet, which described 30.95% of the total variance. Confirmatory factor analysis showed that the 5-factor model was the best fitting model to explain the data.

**Conclusion:**

Considering the need for a tool about in irrational food beliefs, this tool was unable to explain all these dimensions well. It is recommended to create a new questionnaire for the Iranian culture.

**Supplementary Information:**

The online version contains supplementary material available at 10.1186/s12888-023-04909-3.

## Background

Beliefs are thoughts that are determinants to interpreting the events and the quality and quantity of people’s behaviors and feelings. People’s beliefs play an important role in their physical and mental health [1, 2]. Irrational beliefs are non-empirical and impractical and can cause the emergence of important pathogenic events and can affect the quality and quantity of people’s behaviors and feelings. People’s beliefs are important to physical and mental health [1, 2]. These are illogical attitudes and thoughts that have no scientific or practical basis and lead to negative consequences [3]. Beliefs are different in content and form, and they tend to prevent the logical reasoning process [4]. Many of these irrational beliefs are rooted in traditional and cultural beliefs and are accepted as a truth as part of our culture [5].

In cognitive behavioral therapy, irrational beliefs are thought to be the leading cause of psychopathology, including eating disorders and addictive behaviors like food addiction. Albert Ellis and Aaron T. Beck reported that people’s habits usually come from a series of fixed thought patterns that Ellis called irrational beliefs that can lead to ineffective emotional or behavioral reactions. These illogical beliefs originate from the main process of perfectionism or absolutist thinking. According to Beck, people’s emotional responses are consistent with their distorted evaluation of events and not reality. For example, when a distressing event happens to a person, it does not make them anxious, but their perception of that event leads to distress [6, 7].

People with irrational beliefs think that the worst has happened when they make a mistake, and they feel distressed; on the other hand, they attach great importance to the approval of others. This absolutist thinking leads to anxiety, and consequently, one looks for irrational coping strategies, such as drug use and excessive eating (emotional eating) [8–11].

One of the irrational beliefs is having an irrational belief about food. Having false and illogical beliefs acts as knowledge; unhealthy eating and behaviors around food. Irrational food beliefs as a cognitive mechanism can play a role in eating behaviors and weight changes. Because these beliefs are distorted, unhealthy food-related beliefs undermine success in losing and maintaining weight [12]. In addition, irrational belief predicts thinness, dissatisfaction with the body, inefficiency and ignorance in eating. Similarly, studies showed that irrational belief predicts obsession with eating, dieting and fear of obesity [13, 14]. It should be noted that the wrong eating pattern occurs not only individually but is also observed in the family and social environment [15]. Studies indicate that behavioral patterns in families may lead to the choices about food and health. The family-oriented approach emphasizes how family members treat, communicate, and support each other regarding eating behavior and lifestyle changes [16]. Studies have indicated that emotional eating often happens in obese populations, so that overweight people usually report more involvement in emotional eating than people with healthy weight [17]. Obese people have irrational beliefs due to unrealistic expectations and not accepting themselves. As a result, they mostly use ineffective emotions, such as eating food and snacks, when faced with environmental stressors instead of solving the problem, resulting in weight gain [18, 19].

It should be noted that the relationship between mental health and eating habits is two-way phenomenon, so that mood affects eating habits, and eating habits affect mood and mental health [20]. Changes in irrational beliefs is accompanied by reducing different clinical situations, including anxiety and depression [21]. Irrational beliefs resulting from pessimism and absolutist thinking are known as the major cause of mental health, such as anxiety, depression, anger, and loneliness, and subsequently lead to dysfunctional behaviors, such as eating unhealthy foods, overeating, and an eating disorder, and drug use [22, 23]. Anxiety increases appetite and leads to a one desire for fatty and unhealthy foods and sweet substances, or it can cause overeating as a coping strategy [24]. It has been proven that obese people suffer from high negative mood, when a person believes that eating improves mood, he turns to eating in response to negative emotions to regulate negative mood [25]. False beliefs, such as low confidence in the ability to make changes (low self-efficacy). Furthermore, false beliefs that external factors control health (external source of control), and misbeliefs with undesirable outcomes (unfavorable consequence expectations) can be effective in the occurrence of unhealthy nutrition and low physical activity [26].

Due to the importance of the adverse effects, many studies have been conducted to identify factors contributing to irrational food beliefs, and researchers have attempted to measure these beliefs. Tools such as the Dichotomous Thinking in Eating Disorders Scale (DTEDS) [27], the Eating Disorder Core Beliefs Questionnaire (ED-CBQ) [28], the Eating Disorder Belief Questionnaire [29], and the Food Thought Suppression Inventory (FTSI) [30] have been used in some studies. The above-mentioned tools have many limitations such as in their subscales for detecting irrational food beliefs. Osberg et al. (2008) conducted a study entitled “The Irrational Food Beliefs Scale: Development and validation” [31].They believe that irrational food beliefs are defined as distorted or unrealistic attitudes or beliefs about food, which are examples of irrational food beliefs, such as “food is my only source of pleasure” or “life is not worth without ice cream”. It is believed that irrational food beliefs often prevent a person in succeeding with weight control. Thus, a questionnaire containing 57 items was designed to avoid complications. As some researchers pointed out in the process of conducting their research, that large number of phrases in a tool can prevent the participants from answering questions or phrases correctly. Besides, considering the importance of a reliable, valid, and applicable tool is necessary to identify irrational food beliefs. Thus, sub-scales of these beliefs should be identified, and the necessary interventions should be carried out to prevent diseases. Since there are many differences between the customs of European and Asian people in terms of architectural style, dressing, rules and access to food, and mainly due to the geographical location and climate, they have different nutritional styles [32], and in Iran, a specific and suitable tool with the above conditions that can identify the mentioned items has not been used so far; Hence, the purpose of the present study is the translation and psychometric analysis of the Irrational Food Belief Scale proposed by Osberg in order provide a standardized and reliable tool by Iranian culture and health system.

## Methods

This study was carried out in two stages of translation and psychometric analysis of the tool, and the results of each section are explained separately.

### Step 1: translation of the tool

**Fig. 1:**
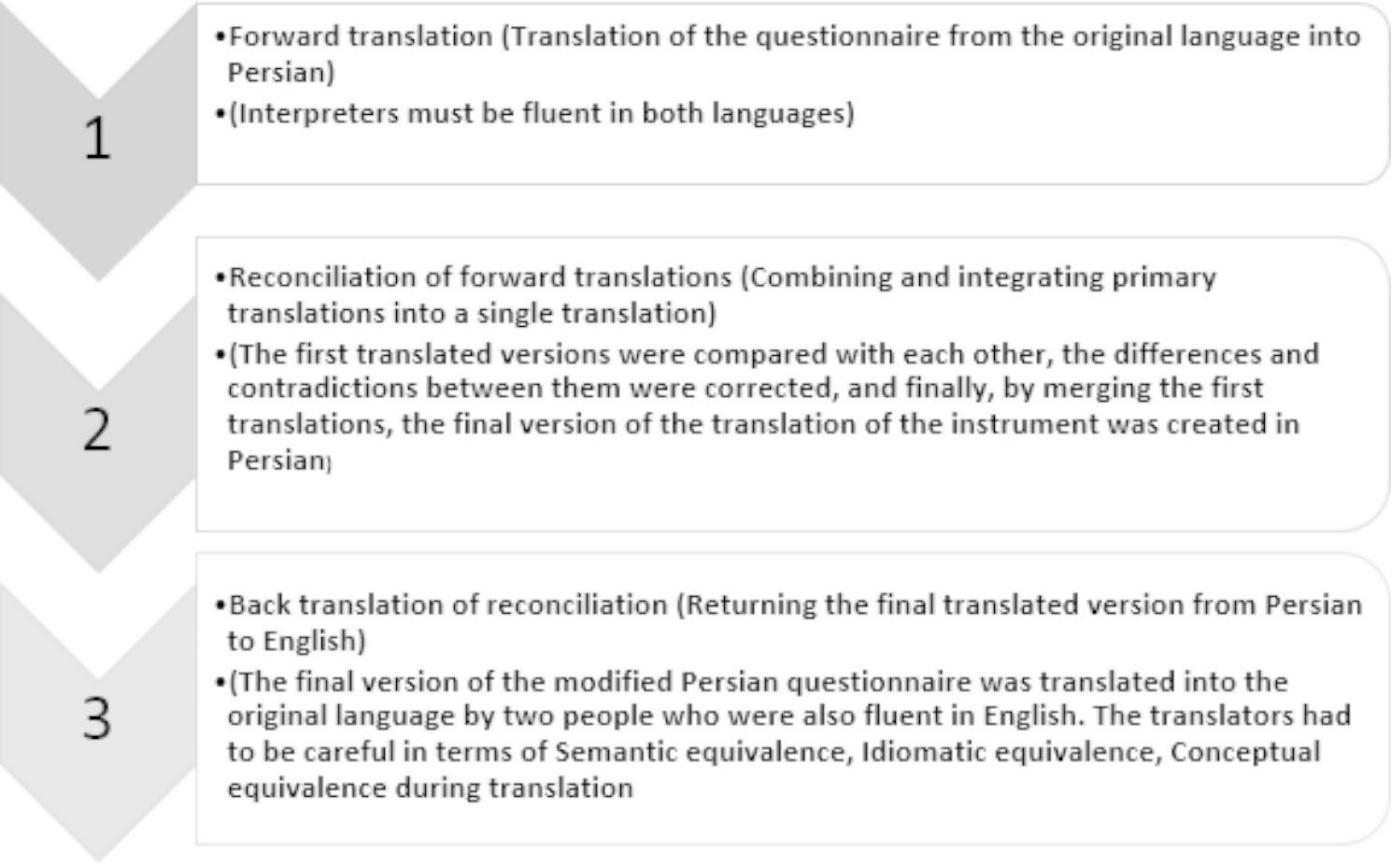
The flowchart of translation of irrational food beliefs

After obtaining written permission from the designer of the scale, the researcher carried out the translation process [33, 34]. For translation, forward backward method of translation was used. First, two translators fluent in Farsi and English translated the questionnaire into Persian. Then, the two translations were combined, and the best translation was selected for each item. Then the designed Persian questionnaire was translated into English by two translators unfamiliar with the original questionnaire. After checking, revising, correcting, and merging these two translations, the new version was sent to the questionnaire’s designer and the items were approved in terms of the same meanings (Fig. 1).

### Step 2: psychometric analysis of the tool

Face validity, content, and construct validity (exploratory, confirmatory factor analysis) were used to check the psychometric properties of the tool. Also, Cronbach’s alpha coefficient, Average Inter-Item Correlation (AIC) and McDonald’s Omega, composite validity, maximum reliability H were used to test the reliability of the tool.

#### Face validity

Face validity was checked by qualitative and quantitative methods in 15 people. For the qualitative face validity, 15 people were surveyed on 57 items of the scale, and at the end, people were asked about difficulty, irreverence, and ambiguity in answering the question. Based on the participants’ opinions the items were revised and modified by the research team, and no items were deleted.

Then quantitative face validity was checked. The impact score of each item was calculated based on its importance, using the impact score formula (Impact Score = Frequency (%) × Importance). The same participants were asked to rate the importance of the tool’s items on a 5-point Likert scale, ranging from 1 (unimportant) to 5 (very important). Then, the impact score of each item was calculated by multiplying the frequency of participants who gave that item a score of 4 or 5 by the average importance score of that item. Items with an impact score > 1.5 were considered acceptable [35, 36].

#### Content validity

Content validity was checked by 15 experts using both qualitative and quantitative methods. For the qualitative content validity, 15 qualified experts (five clinical psychologists, four psychometric and methodology experts, two health psychology experts, four nutritionists) were interviewed. Regarding the difficulty level, literature, simplicity, fluency and comprehensibility of the irrational health belief scale items, the experts recorded their opinions in writing for the researcher. Then, the researcher applied the experts’ suggestions to the scale.

In the quantitative part of content analysis, content validity ratio (CVR) and content validity index (CVI) were calculated for the items. First, the experts were asked to identify the necessity of each item on a 3 ranges scale: (3 = essential, 2 = useful but not essential, and 1 = not essential. Then, CVR was calculated based on the formula CVR= (Ne-N/2)/N/2. Finally, the resulting number was compared with Lawshe table. It was considered acceptable if the value obtained from the CVR based on the Lawshe table was 0.49 or more (for 15 experts). Thus, it shows that the presence of the relevant term with a statistical significance level (p < 0.05) is important in this tool [35].

CVI was used to indicate the relevance of the items. The items were evaluated on a 4-point Likert scale: (1 = not relevant, 2 = somewhat relevant, 3 = completely relevant, and 4 = very relevant). The number of experts who gave the item a score of 3 or 4 was divided by the total number of experts, and also a value between 0 and 1 was obtained. If the score content validity index was higher than 0.79 that item was considered acceptable and remained in the questionnaire. If the content validity index score is between 0.7 and 0.79, the item needed to be revised and corrected. A content validity index score of less than 0.7 is considered unacceptable and would be removed [37].

The modified Kappa statistic was determined for each item to consider the expert panel’s chance agreement. Items with a Kappa value of 0.7 or more were considered appropriate [36].

#### Construct validity

Exploratory factor analysis (EFA) and confirmatory factor analysis (CFA) were used to determine the construct validity of the tool. According to Monro, 500 samples for exploratory factor analysis (5–10 subject per item) and 500 samples for confirmatory factor analysis (5 to 10 subject per item) are considered suitable [38, 39]. In the first step, latent variables were extracted using EFA and SPSS 28. The sampling adequacy index was calculated with Kaiser-Meyer-Olkin (KMO) and Bartlett’s tests. A KMO value greater than 0.7 was interpreted as a sufficient sample [40]. Next, the latent factors were extracted using Maximum likelihood, Promax rotation, and Horn’s Parallel Analysis [41]. The criterion for selecting items was a factor loading above 0.3, and Eigenvalues ​​greater than one were found to be suitable for extracting factors [42].

Online data were collected using a demographic questionnaire and the Irrational Food Belief Scale (IFBS) for exploratory factor analysis. The research was carried out after obtaining ethics approval from the Ethics Committee of Azad University of Medical Sciences. The inclusion criteria were being over 18 years old, and being proficient in reading and writing. In the next step, the fit of the factors extracted in the first step was checked by CFA and AMOS 26. Finally, the extracted factors were confirmed based on model fit indexes (Table 1).

**Table 1:** Fit model indices of irrational food beliefs scale

Model\Indices	χ^2^	df	P value	CMIN/DF	RMSEA	AGFI	PNFI	IFI	CFI
**First-order**	1316.13	314	0.001	2.471	0.054	0.872	0.712	0.95	0.95
**Cut off**	-	-	> 0.05	< 3	< 0.08	> 0.5	> 0.5	> 0.9	> 0.9

**χ**^**2**^ **=** Chi-squared, **df =** Degrees of Freedom, **CMIN/DF =** Minimum Discrepancy Function by Degrees of Freedom Divided, **RMSEA =** Root Mean Square Error of Approximation, **AGFI =** Adjusted Goodness of Fit Index, **PNFI =** Parsimonious Normal fit Index, **IFI =** incremental fit Index, **CFI =** Comparative Fit Index

#### Reliability

The reliability of the tool was calculated using Cronbach’s alpha (0.7 < acceptable), McDonald’s Omega (0.7 < acceptable), and inter-item correlation (AIC between 0.2 and 0.4) [43].

## Results

The average age of the participants’ was 37.88 (SD ± 11.47) years, BMI was 25.65 (SD ± 4.22). 55.8% of the participants were women, and 45.3% were in the normal weight range and 54.7% were overweight or obese. Also, 46.1% of the samples in this study had a high school diploma or less, and 53.9% had earned a higher education level. 79.1% were healthy and the rest had chronic diseases. First, qualitative Face validity was access. Then, five items were revised because some words were incomprehensible for the target group. For example, in the sentence “Unsaturated fat is better than saturated fat”,“ the term “unsaturated fat” was not understood by the target group. Therefore, this sentences changed to “liquid oil is better than solid oil”. Then, the evaluation of the quantitative face validity showed that the impact scores of the items were between 2.38 and 6.5, and therefore none of the items were removed.

For qualitative content validity, 15 qualified experts (five clinical psychologists, four instrumental and methodology experts, two health psychology experts, four nutritionists) were surveyed in writing, and five items were modified. CVR is a number between 1 and − 1. Lawshe’s table determines the minimum CVR. According to Lawshe’s Table (15 experts) CVR must be higher or equal to 49% to be considered acceptable, and items less than this number must be removed [44]. In this research, the total CVR was 0.91% and varied between 0.6 and 1 for each item. Therefore, no items were removed at this stage. The CVI for the whole scale was calculated to be 0.91, which ranged from 0.86 to 1 for each item, and since CVI > 0.79 is considered appropriate [45], all the items were approved and no items were deleted. In EFA, the Kaiser-Meyer-Olkin index was 0.91, and the value of Bartlett’s test was 7559.88 (p < 0.001). In the exploratory factor analysis, 30 items were removed and the remaining 27 items were loaded on five factors, which described 30.95% of the total variance. These five factors included behavioral and psychological aspects, nutritional attitudes, healthy eating, control eating, and diet (Table 2).

**Table 2:** The results of Exploratory Factor Analysis of Irrational Food Beliefs Scale

Factors	Items	Factor loading	h2	% of variance	Eigenvalues
Behavioral and psychological aspects	Q10: Food is a good way to eliminate depression.	0.771	0.594	10.866	2.934
	Q7: Eating is a good way to overcome boredom.	0.719	0.516		
	Q16: Eating is an appropriate way to decrease stress	0.668	0.446		
	Q55: Eating can help overcome loneliness.	0.654	0.427		
	Q44: Happiness can be achieved by eating.	0.597	0.356		
	Q6. My greatest pleasure in during life is eating food.	0.534	0.285		
	Q30: Foods should be the major part of all social meetings.	0.402	0.161		
	Q11: Social events are not very attractive without food	0.387	0.149		
Nutritional attitudes	Q45: If the food is low fat, you can eat as much as you want.	0.696	0.484	8.066	2.178
	Q36: Foods that you eat before 8 pm, do not cause the overweight.	0.639	0.408		
	Q37: If I exercise first, I can eat whatever I want.	0.562	0.315		
	Q19: If something is fat free, you can eat as much as you want of it.	0.542	0.293		
	Q39: Foods such as fruits and vegetables have no calories.	0.523	0.273		
	Q57: If you exercise, not matter what you eat.	0.476	0.226		
	Q38: Overweight is genetic so why I bother myself to lose weight?	0.424	0.179		
Healthy eating	Q29: Eating healthy can reduce risk for some diseases such as cancer, diabetes, and heart disease.	0.634	0.401	4.959	1.339
	Q17: The key for a healthy diet is to achieve a balance in the foods that you eat.	0.566	0.320		
	Q12: Eating healthy should be considered as the life style	0.488	0.238		
	Q49: What a person eats really has no effect on their health.	0.481	0.231		
	Q33: Food is an alternative way for sex.	0.387	0.149		
Control eating	Q26: There are foods that I cannot control my intake.	0.714	0.509	4.529	1.223
	Q25: Because I love eating, I really cannot control my weight.	0.690	0.476		
	Q27: I should eat sweets to stay alive.	0.362	0.131		
	Q32: Eating is my pleasure and I should not regulate the consumption of foods.	0.328	0.107		
Diet food	Q52: Diet food is boring	0.659	0.434	3.525	0.952
	Q51: Diet means giving up eating.	0.564	0.318		
	Q54: Not eating what you want makes you sad.	0.448	0.200		

h^2^ = Communalities

**Fig. 2:**
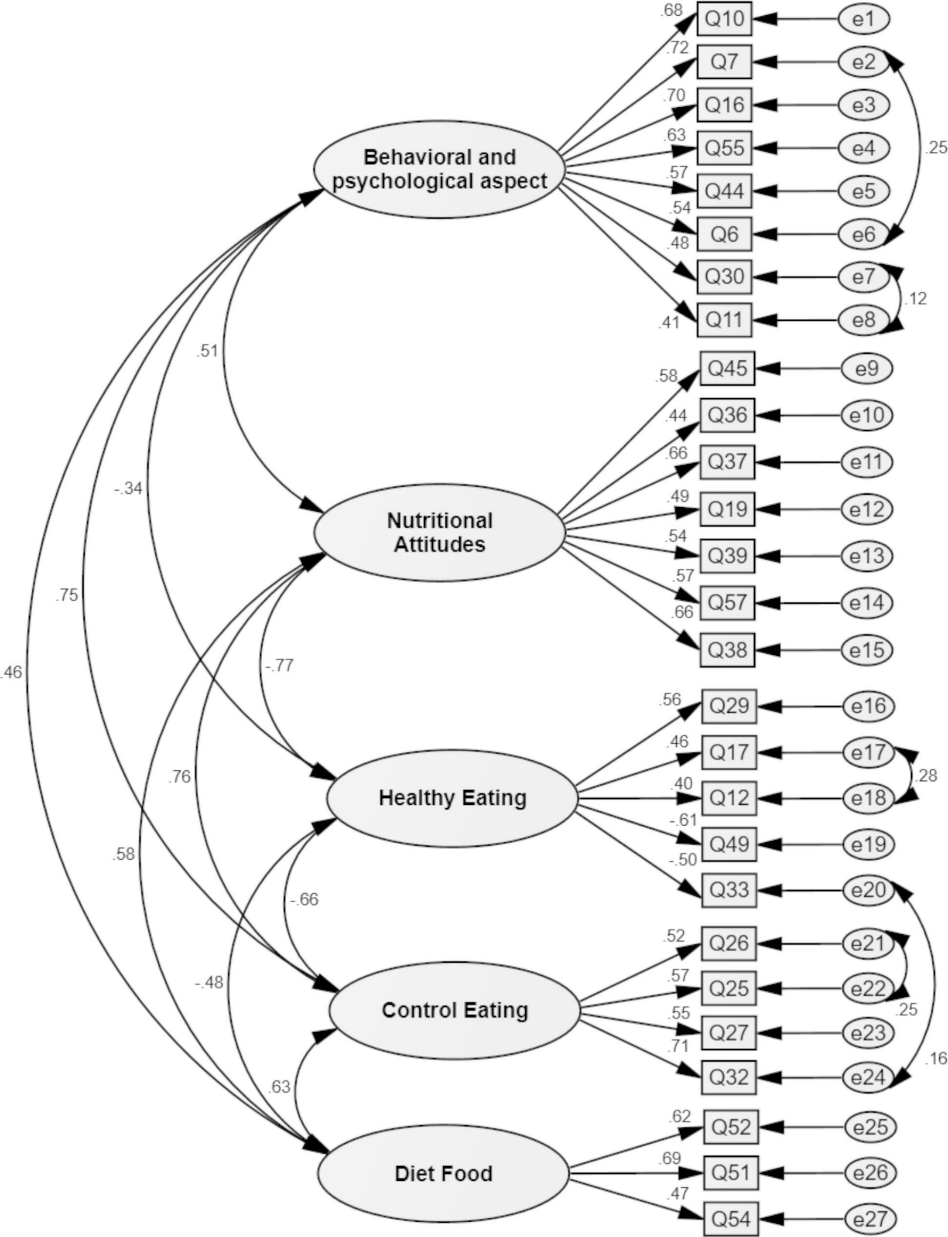
The final structure of irrational food beliefs scale confirmed in CFA (n = 500)

Confirmatory factor analysis showed an acceptable fit. The results of confirmatory factor analysis are shown in Table 1. These findings confirmed the five-factor structure of the 27-item Irrational Food Beliefs Scale. Figure 2 shows this structure and the coefficients of the pairwise correlations between the items and dimensions of the Irrational Food Beliefs Scale.

### Reliability

Internal consistency was obtained as 0.849 through Cronbach alpha. The relative stability of the tool was obtained as 0.92 based on the ICC result (CI 95: 0.85 to 0.95, p < 0.001) (Table 3).

**Table 3:** The reliability of irrational food beliefs scale (subscales)

Factor	Reliability		
	AIC	Omega	Alpha
**Behavioral and psychological aspects**	0.817	0.821	0.358
**Nutritional attitudes**	0.786	0.793	0.347
**Healthy eating**	0709	0.712	0.384
**Control eating**	0.717	0.719	0.390
**Diet food**	0.701	0.702	0.357

## Discussion

This methodological study evaluated the psychometric properties of the Irrational Food Beliefs Scale. In this study, five factors, including “psycho-behavioral aspects”, “nutritional attitudes”, “healthy eating”, “control eating” and “diet” were identified for the tool.

The factors of the Irrational Food Beliefs Scale are discussed below. The factor of psycho-behavioral aspects includes eight items that account for the highest percentage of variance and refers to the amount of pleasure in eating, the effect of eating on overcoming depression, boredom and loneliness, and the effect of food on social gatherings. In a study by Konttinen demonstrated that depression can lead to emotional eating, which affects food choices. Depressed people who suffer from emotional eating eat more sugary foods, while depressed people who do not suffer from emotional eating eat fewer fruits and vegetables. Therefore, both depression and emotional eating affect food choices and increase the consumption of unhealthy foods [46]. Certain foods, especially those with high fat or sugar, may induce “addictive” behavior by causing neurological changes under certain conditions. These consumption patterns are associated with increased risks of diseases such as obesity, early weight gain, depression, anxiety, substance abuse relapse and treatment problems [47]. It is worth noting that Al-Ammar et al. showed that different food choices can enhance or reduce mood. The role of mood in personal food choices, appetite, and desire to eat cannot be underestimated. Consuming healthy foods like vegetables, fruits, protein, nuts, adequate water, and moderate amounts of caffeine can enhance a good mood. Nonetheless, indulging in unhealthy food choices such as excessive chocolate, sweets, fast food, and low-nutritional foods may boost mood. Yet, this effect is transient and can harm one’s health, leading to chronic diseases. People who are stressed may gravitate towards unhealthy foods to improve their mood, but this can ultimately have a negative impact on their mental and physical well-being. Advertising can also play a role in promoting unhealthy food choices that offer a temporary mood boost and energy [48]. Given that Osberg et al. employed obese people to prepare the Irrational Food Beliefs Scale, the highest factor load in irrational food belief has been explained in the “psycho-behavioral aspects” factor, indicating the effects of these factors on the way of eating and choosing daily food.

The factor of nutritional attitudes has seven items and deals with people’s thinking about calories and fat in food and about exercise and its effect on eliminating calories and food fat. Attitude means relatively stable feelings, inclinations or a set of beliefs directed towards an idea, situation, person, or object. Negative attitudes towards obesity and obese people are created through the prevalence of false beliefs [49]. To predict the calorie intake of people in their study, Shah et al. showed that participants who perceived themselves to be overweight followed exercise guidelines and or the healthy food choices and consumed fewer calories than participants who did not have the guidelines. The results showed that education about nutrition, exercise and understanding weight before ordering food leads to better food choices [50]. Studies have shown that home cooked meals are associated with better diet quality [51]. Another study found barriers that low-income Americans face with preparing healthy meals, including lack of access to grocery stores, lack of time to cook, and difficulty in preparing fresh, perishable or unprocessed ingredients [51]. Osberg et al. described two factors of irrational food belief and rational food belief in their scales. Also Lobera et al. mentioned one of the sub-scales of irrational food belief as cognitive distortions and unhealthy beliefs and attitudes related to food [52, 53].

The factor of healthy eating includes five items that address healthy eating, balance, healthy eating style and food replacement with sex. Jaiyungyuen et al. showed that increasing food knowledge, changing people’s beliefs about unhealthy foods, and attracting companionship of influential people could be effective in developing healthy eating habits and sticking to them because family support or companionship are one of the factors contributing to people’s adherence to diet plans [54]. Certain fruits or vegetables may have benefits against certain cancers. There is significant epidemiological evidence that consistently supports the benefits of increased fruit and vegetable consumption to reduce the risk of cardiovascular disease. Also, several studies have shown a relationship between regular nut consumption and lower risks of cardiovascular disease and type 2 diabetes, he because nuts may be rich in unsaturated fatty acids. Substituting protein or unsaturated fat for carbohydrates lowers blood pressure and improves blood lipids [55]. In their scale, Lobera et al. also refer to beliefs that are compatible with current guidelines on healthy eating [53].

The factor of control in eating has four items, which include not controlling eating, the pleasure of eating sweets, and the pleasure of eating. Studies show that people who prefer a stronger sweet taste, compared to those who prefer a weaker sweet taste are likely to score more in the two factors (impaired control over eating sweet foods) and (sensitivity to the mood-altering effect of sweets) [56]. Another study indicated that the perception of pleasure as an obstacle to healthy eating is significantly related to not complying with the recommended consumption of fruits and vegetables for men and women [57]. This sub-factor is also included in the scales of Osberg et al. et al. and Loberta et al. in the irrational health belief factor [52, 53].

The diet factor contains three items related to the attitude towards the diet and the boringness of the healthy diet. Epidemiological studies concerning diet and non-communicable diseases often focus on the role of consuming only one nutrient and the risk of disease. However, given that most foods contain many nutrients and the intake of one nutrient is associated with the input of other nutrients, researchers have focused more on the relationship between diet and the risk of diseases [58]. Furthermore, studies have shown that improving physical access to supermarkets is only sufficient to enhance diet quality if socioeconomic and psychosocial determinants (e.g., prioritizing the importance of healthy eating and providing socioeconomic support for such priorities) are also considered. Similarly, people with a positive attitude towards healthy eating were committed to higher quality diets at any level and cost to purchase healthy foods. Hence, ensuring economic access to healthy foods and motivating consumers to search for healthier food choices through ongoing nutrition education are equally important strategies for improving diet in the population [59].

## Conclusion

In this study, Osberg’s Irrational Food Beliefs Scale was evaluated, and also the five factors of “psycho-behavioral aspects”, “nutritional attitudes”, “healthy eating”, “control in eating” and “diet” were identified for the tool. These sub-scales have 27 items that explain 30.95% of the concept. However, irrational food beliefs have psychological, behavioral and attitudinal aspects regarding nutrition and are related to the culture of the society and its food-related habits, Therefore, this scale cannot express a complete understanding of this factor in Iranian culture, Because in Iran, there are different food cultures and beliefs according to the country’s dominant religion. Culture of any country determines people’s attitudes and opinions about the food they eat. Beliefs determine people’s lifestyles, what they eat, and what religion they follow. Therefore, we recommend that a qualitative research study to be conducted to define the concept in our society, produce its items, convert it into a tool and psychometrically test it so that a valid and reliable tool can be used in Iran.

### Study limitations and suggestion

The most important limitation of this study is that the researchers could not take samples from different cultures that have different nutritional habits. Since there are different ethnicities in Iran and nutritional beliefs are different, also as the beliefs about food in Iran are different from other countries in terms of the dominant religion of the country and the choice of type of food, therefore, a tool with Iranian culture and beliefs is needed.

## Electronic supplementary material

Below is the link to the electronic supplementary material.

Supplementary Material 1

## Data Availability

The datasets generated during the current study are not publicly available; however, reasonable data request will be reviewed and granted by the corresponding author.

## Abbreviations

CVR: Content validity ratio
CVI: Content validity index
KMO: Kaiser-Meyer-Olkin
MLE: Maximum likelihood exploratory
GFI: Goodness of fit index
RMSEA: Root mean square error of approximation
CMIN/DF: Chi-square mean/degree of freedom
IFI: Incremental fit index
