# Supplementary material for: The translation and psychometrics Persian version of irrational food belief scale
Source: BMC Psychiatry. 2023 Jun 15;23:435. doi: 10.1186/s12888-023-04909-3 (PMC10268467; doi:10.1186/s12888-023-04909-3)
Supplement: Supplementary file 1 — Supplementary Material 1 [file 12888_2023_4909_MOESM1_ESM.docx]

**Appendix**

Irrational food beliefs scale (English version)

1. Food is a substitute source of comfort.

2. Some foods are able to relax you.

3. Eating healthy doesn't take more time than unhealthy eating.

4. I can't possibly live without my favorite food.

5. Broiling and roasting meats is a healthy way to cook them.

6. My greatest pleasure in life is eating.

7. Eating is a good way to overcome boredom.

8. Exercise can undo the effects of a poor diet.

9. Eating healthy does not have to mean giving up my favorite foods entirely.

10. Food is a good way to lift depression.

11. Social events are not as fun without food.

12. Healthy eating should be a way of life.

13. If no one sees me eating something, the calories don't count.

14. Only high fat foods taste good.

15. The only way to diet is to crash diet.

16. A good means of stress reduction is to eat.

17. The key to a healthy diet is to achieve balance in the foods you eat.

18. Some foods are irresistible.

19. If something is fat free, you can eat as much as you want of it.

20. Unsaturated fat is better than saturated fat.

21. Breakfast is the most important meal of the day.

22. If you eat something you shouldn't, you should feel guilty.

23. There are some foods you can have in an unlimited amount and not gain weight.

24. One should strive for 5 servings of fruits and vegetables a day.

25. I simply cannot control my weight because I love to eat.

26. There are some foods over which I cannot control my intake.

27. I must have sweets to exist.

28. it's important to have at least 6 servings a day of the food group that includes bread, cereal, rice, or pasta.

29. Eating healthy can reduce risk for some diseases such as cancer, diabetes, and heart disease.

30. All social gatherings must be centered on food.

31. Some foods are addictive.

32. Food is my one pleasure and I should not have to regulate my intake of it.

33. Food is a good substitute for sex.

34. To hell with what's healthy, let me eat what I want.

35. Calcium enriched foods are needed for strong bones.

36. You won't gain weight for anything you eat before 8 p.m.

37. If I exercise first, I can eat whatever I want.

38. Being overweight is genetic, so why bother trying to lose weight?

39. Foods like fruits and vegetables have no calories.

40. There are times when I NEED certain foods.

41. One should choose lean or low-fat meats.

42. You can drink as much of fluids as you want and not gain weight.

43. A small amount of fat is needed in a healthy diet

44. Happiness can be achieved through eating.

45. You can eat as much as you want as long as it's low fat.

46. Once you eat something bad, you've blown your diet.

47. I believe it is important to eat only when you are hungry.

48. Because alcohol has no fat, it can't make you gain weight.

49. What a person eats really has no effect on their health.

50. It is punishment to have to eat certain foods like fruits and vegetables.

51. To diet is to give up the pleasure of eating.

52. Diet food is boring.

53. One should strive to eat 3 healthy meals a day.

54. Not being able to eat what you want will make you sad.

55. Eating can help overcome loneliness.

56. I believe in the food pyramid as a guide to healthy eating.

57. If you exercise, it doesn't matter what you eat.
